# The Role of Cellulose Charge and Matrix Composition on KNO_3_‑Nutrient Release Kinetics and Mechanisms

**DOI:** 10.1021/acsomega.5c06715

**Published:** 2026-02-19

**Authors:** Débora França, Sahmira Bianchi, Roselena Faez

**Affiliations:** † Laboratory of Polymeric Materials and Biosorbents, Universidade Federal São Carlos, UFSCar, Rod. Anhanguera, km 174, Araras, SP 13600970, Brazil; ‡ Graduate Program in Materials Science and Engineering, Universidade de São Paulo, USP- FZEA, Pirassununga, SP 13635900, Brazil

## Abstract

The global demand for food is driving research into regenerative agriculture, particularly the development of enhanced efficiency fertilizers (EEFs) that aim to improve nutrient use efficiency and reduce environmental impact. Understanding the release mechanisms of EEFs is essential for designing more effective formulations. This study examines the nutrient release kinetics of EEFs, focusing on potassium nitrate (KNO_3_) embedded in a cellulose-based matrix produced via spray-drying and melt-processing. Several mathematical models – Zero-order, First-order, Higuchi, Korsmeyer-Peppas, Peppas-Sahlin, Hixson-Crowell, and Hopfenberg – were used to analyze nutrient release dynamics in water. The results showed that the matrix structure, particle formation method, and CNF charge influence the release mechanism. The Korsmeyer-Peppas and Peppas-Sahlin models best described the anomalous release behavior. K^+^ release is primarily driven by diffusion, and NO_3_^–^ release is governed by matrix relaxation when combined with positively charged CNF (CNF^+^). In contrast, K^+^ is released via matrix relaxation and NO_3_^–^ via diffusion when combined with negatively charged CNF (CNF^–^). Adjusting CNF charge or matrix composition can optimize nutrient release, improve fertilizer management, and enhance sustainability in agricultural practices.

## Graphical abstract

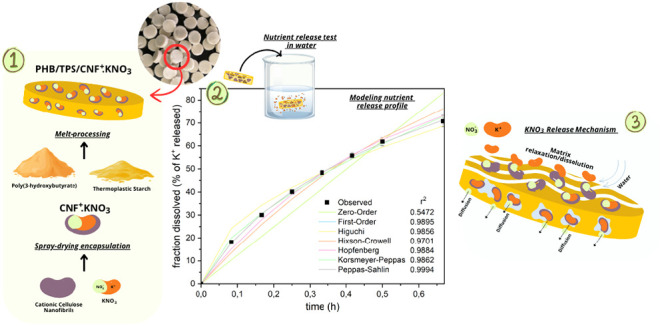

## 1 Introduction

The need for regenerative agriculture practices to achieve the Sustainable Development Goals (SDGs) of zero hunger and responsible consumption has intensified research on enhanced efficiency fertilizers (EEFs).^1^ Rising food production to meet population growth requires greater fertilizer and water inputs, yet the rapid dissolution of conventional fertilizers limits nutrient uptake, impairing soil and water quality.^2^ EEF mitigates this by supplying nutrients gradually, reducing application frequency, and lowering nutrient losses.^3^ Although fertilizer coatings based on petroleum-derived synthetic polymers with better NUE are already commercialized, they cause the accumulation of microplastic residues in the soil.^4^ Selecting an appropriate matrix for EEFs requires both sustainability and efficacy, and biopolymers fulfill these criteria. Several studies using lignocellulosic materials and polysaccharides, such as cellulose, lignin, starch, and chitosan, have demonstrated the effectiveness of applying biopolymers in EEFs.^5–7^

Understanding nutrient release behavior from biopolymer-based EEFs is essential for optimizing their performance. As in drug delivery systems, release is governed by processes such as dissolution, diffusion, swelling, and erosion,^8,9^ with water-EEF interaction as the primary factor.^12,13^ Matrix composition and structure exert a strong influence through properties such as solubility, degradation rate, particle size, and molecular weight.^10,11^ In the context of EEF, nutrient release is influenced by multiple factors - such as soil pH, exchange capacity, moisture, organic matter content, compaction, microbial activity, root architecture, temperature, and light. Therefore, nutrient release tests conducted in water reflect the effects of water (and its pH) alone, which can help predict and better understand the release pattern of the same EEF in soil conditions. Angelo et al.^14^ provide examples comparing water and soil experiments. For their chitosan-based EEF, an hour release in water corresponds to a 20-day release in soil under tropical conditions.

Mathematical modeling is a powerful tool for describing, predicting, and optimizing nutrient release from EEF. Experimental verification complements modeling by elucidating release mechanisms and their dependence on time.^15^ Models may represent physical phenomena or empirical, derived from experimental data.^8^ Table 1 shows some of the mathematical models that describe the controlled release of active agents, and their respective equations. Key parameters commonly used include *F* (amount of nutrients released), *F*_0_ (initial amount), *F*_*i*_ (remaining at time *t)*, and *k* (model-specific constant). Zero-order kinetics assumes a constant release, independent of active agent concentration.^16^ First-order kinetics describes release proportional to the remaining nutrient in the matrix, commonly applied to soluble active.^17^ Higuchi model^18^ represents diffusion-controlled release from homogeneous matrices with assumptions about initial concentration, diffusion type, and matrix geometry.^19,20^ Hixson and Crowell^35^ relate release to changes in surface area and volume, useful for matrices that dissolve while maintaining the shape.^16^ The Hopfenberg model^21,22^ addresses release from surface-eroding polymers, with geometry-dependent parameters (*n* = 1 for film, 2 for a cylinder, and 3 for a sphere).

**Table 1:** Mathematical Models and Their Corresponding Equations Describe the Controlled Release of Active Agents

model	equation	
Zero-order	*F* = *F*_0_ + *k*_0_ *t*	Dash et al.^b^ ^33^
First-order^a^	$\frac{\mathrm{dF}}{\mathrm{dt}}=-\mathrm{kt}$	Noyes and Whitney^34^
Higuchi	*F* = *k*_*H*_ *t* ^0.5^	Higuchi^18^
Hixson-Crowell	$\sqrt[{3}]{F_{0}}=\sqrt[{3}]{F_{i}}+k_{\mathrm{HC}}t$	Hixson and Crowell^35^
Hopfenberg^a^	*F* = 100 [1–(1–*K*_*HB*_ *t*)^*n*^]	Hopfenberg^21,22^
Korsmeyer-Peppas	*F* = *k*_*KP*_ *t* ^*n*^	Korsmeyer et al.;^36^ Ritger and Peppas^37,38^
Peppas-Sahlin	*F* = *k*_1_ *t* ^*m*^ + *k*_2_ *t* ^2*m*^	Peppas and Sahlin^39^

a Equations that have changed over time.

b Reference to a study employing this equation.

Semiempirical models, such as Korsmeyer-Peppas and Peppas-Sahlin, are suited for systems where multiple release mechanisms coexist.^23–26^ The exponent *n* indicates transport type: Fickian diffusion (*n* = 0.43), Case-II transport (*n* = 0.89), or anomalous transport (0.43 < *n* < 0.89). The Peppas-Sahlin model separates diffusional (*k*_1_) and relaxational (*k*_2_) contributions, when *m* = *n* relaxational mechanism is negligible (Table 1).

These kinetic models have been applied to EEFs in forms such as granules, films, hydrogels, and pellets, produced via coating, wet-extrusion, cross-linking, and hot-press. Lakshani et al.^27^ compiled examples of a wide range of materials used for slow release of fertilizers, including organic polymers, e.g., chitosan, poly­(vinyl alcohol), lignin, and inorganic compounds, e.g., hydroxyapatite, montmorillonite, bentonite. Among these, hydroxyapatite, chitosan, and starch-based polymers have been extensively studied for their release kinetics using models such as zero-order, first-order, Higuchi, Korsmeyer-Peppas, and Peppas-Sahlin, demonstrating their controlled nutrient release under various environmental conditions.^8^ Most studies focus on modeling urea release from different matrices. For example, Araújo et al.^27^ found Higuchi’s model best described the diffusion-based release of urea from chitosan-coated granules. Shen et al.^28^ reported that urea release from carboxymethyl cellulose hydrogels followed a First-order model, while Ko et al.^29^ observed Zero-order kinetics in rosin-based pellets. Variations in matrix types, testing methods, and environmental conditions significantly influence release behavior, making existing models useful as a reference for similarly formulated EEFs.

For potassium nitrate (KNO_3_), Chiaregato et al.^30^ showed that its release from pellets composed of the salt, sugar cane bagasse, and poly­(3-hydroxybutyrate) aligned with the Peppas-Sahlin. On the other hand, Joshi et al.^31^ found that KNO_3_ and potassium phosphate released from sodium alginate-based microspheres followed Zero-order kinetics.

The present study aimed to apply and evaluate multiple kinetic models (Zero-order, First-order, Higuchi, Korsmeyer-Peppas, Peppas-Sahlin, Hixson-Crowell, and Hopfenberg) to characterize and predict nutrient release in water from a KNO_3_-cellulose-based EEF produced via spray-drying and melt processing. We hypothesize that nutrient release kinetics are influenced by particle morphology and matrix composition, and that different models will capture distinct release mechanisms under varying conditions.^32^ Computerized modeling was used to fit experimental data and determine key kinetic parameters, enabling prediction of the release behavior in similar matrices. Understanding these kinetics is essential for optimizing fertilizer efficiency, minimizing nutrient losses, and guiding the design of formulations for sustainable agricultural practices. Although this study focused on modeling rather than material preparation and characterization, previously published characterization data provide essential support for the modeling approach.

## 2 Experimental Section

### 2.1 Production of Cellulose-Based EEFs

The experimental data from the nutrient release test of the EEFs were used to fit various kinetic models, allowing for the evaluation of the best-fitting model. The EEF used for the experimental assessment was in tablet form, consisting of a solid, biopolymer-based composition. A detailed account of the composition, preparation methodology, and the chemical, morphological, structural, and thermal properties, as well as the soil release and biodegradation tests of the EEFs, is provided in a previous study by França et al.^40^ In summary, the EEFs were tablets composed of functionalized microparticles containing cationic or anionic cellulose nanofibrils (CNF^+^/CNF^–^, 1.22% in microspheres and 10% in microcapsules) and KNO_3_ (48.78% in microspheres and 40% in microcapsules), embedded in a polymeric matrix of poly­(3-hydroxybutyrate) (PHB) with starch (St) or thermoplastic starch (TPS). The microparticles were obtained by spray-drying, resulting in two types: microspheres (using a 2-fluid nozzle) and microcapsules (using 3-fluid nozzles). These microparticles were subsequently inserted into the polymeric matrix via the hot-melting process. Eight EEFs were developed and named according to the matrix, functionalization, and microparticle type (Table 2). Morphological and thermal analysis was conducted using, respectively, scanning electron microscopy (SEM) and differential scanning calorimetry (DSC), with parameters and specifications also detailed by França et al.^40,41^ França et al.^41^ also present detailed information regarding the production, chemical, morphological, structural, and thermal properties of the coating matrices employed in the formulation of the tablets investigated in the present study. (PHB.St and PHB.TPS). It is important to note that, although direct structural confirmation of the microcapsules was not performed in the samples, a recent publication demonstrated the formation of core–shell structures using dye tracer and SEM analysis under the same spray-drying conditions and 3-fluid nozzle parameters.^42^

**Table 2:**
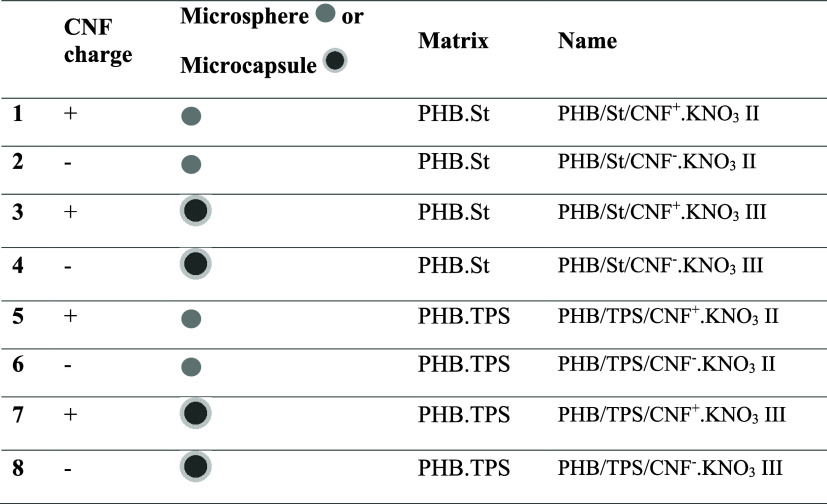
Composition of the Tablets Used for the Nutrient Release Test in Water

### 2.2 Nutrient-Release Test

The nutrient release rate was evaluated as a function of the K^+^ and NO_3_^–^ concentration from KNO_3_ available in water over time. This evaluation was performed in triplicate, following methods used in our previous studies.^43–46^ Tablets (200 mg) were placed in small, permeable sachets (dimensions 7 cm × 5 cm) and submerged in 50 mL of distilled water (25 °C, pH 8 and electrical conductivity approximately 4.6 μS/cm). At a specific time (5/10/15/20/25/30/40/50 min, 1/1.5/2/2/3/4/5/6 h, and at 2/3/4/5/6/7 days) the sachet was removed and submerged in another 50 mL of distilled water (Figure 1e). Potassium ion (K^+^) content was determined using a flame photometer (Digimed – DM 62) and nitrate ion (NO_3_^–^) via UV–vis spectrometer (Thermo Scientific – Genesys 10S UV–vis). Cumulative concentrations of released K^+^ and NO_3_^–^ (in ppm) were calculated over time using the respective calibration curves for each ion. The longest time point measurement was set as 100% to calculate the percentage of nutrient release.

**Figure 1:**
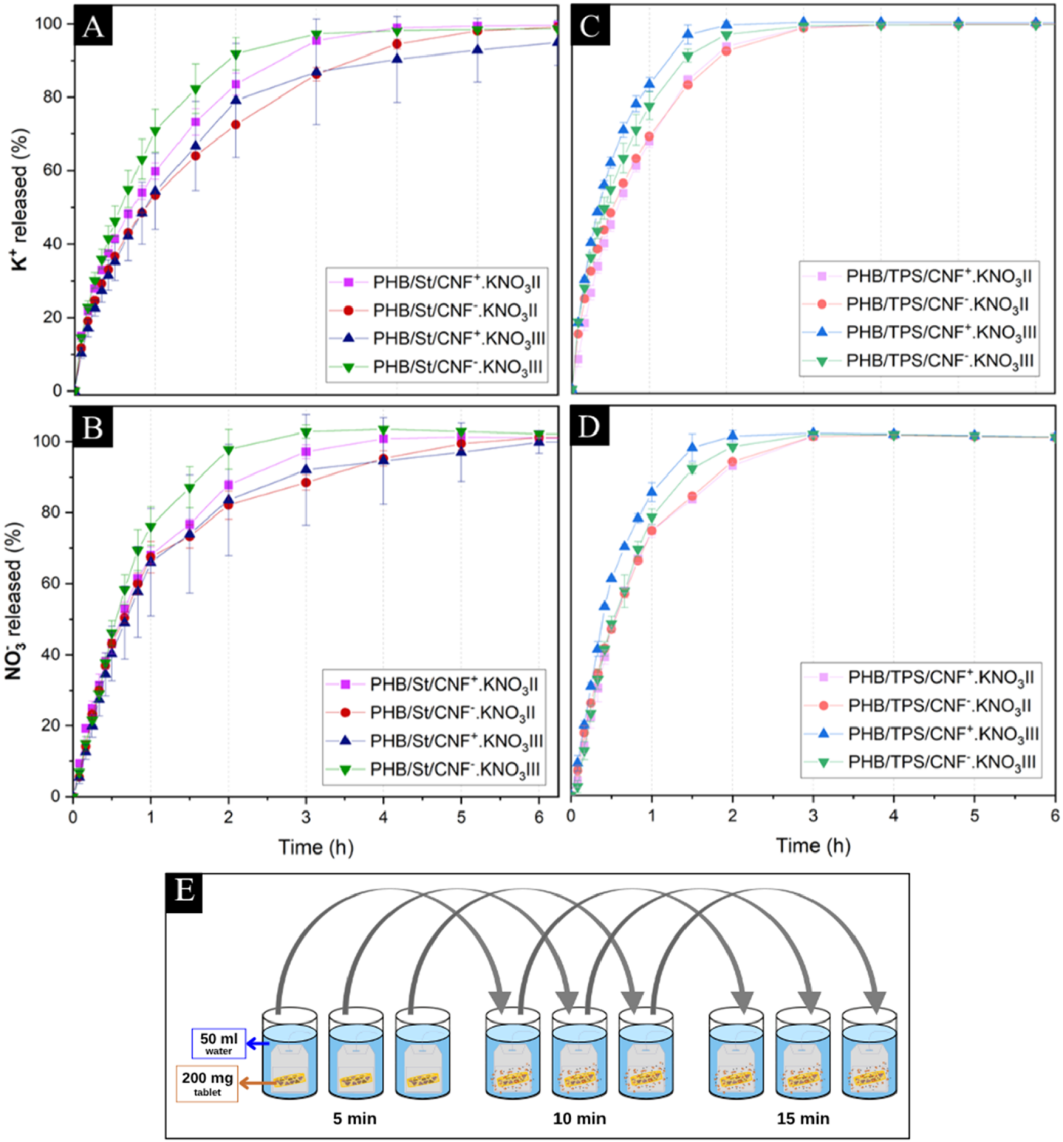
Cumulative potassium and nitrate percentage of spray-dried microspheres and microcapsules with (a, b) PHB.St or (c, d) PHB.TPS matrix and representation of the methodology used for the water release test (e).

### 2.3 Modeling Nutrient Release Data

Seven mathematical models were fitted to the experimental nutrient release data to determine the release mechanisms of KNO_3_ from EEFs: Zero-order, First-order, Higuchi, Korsmeyer-Peppas, Peppas-Sahlin, Hixson-Crowell, Hopfenberg. These models were adjusted to the experimental data to evaluate the nutrient release kinetics using the DDSolver add-in for Microsoft Excel.^47^ To ensure consistent model comparison and because the short-time approximation is valid for the Korsmeyer-Peppas and Peppas-Sahlin models, only the first 60% of the released nutrient was considered. This initial phase also reflects the early availability of nutrients, which is critical for supporting initial plant growth.^39^ We determined the best-fitting model based on a higher coefficient of determination (*r*_adjusted_^2^) and a lower Akaike Information Criterion (AIC).

### 2.4 Swelling Degree

To assess the swelling degree of the matrices used in the composites, tablets made solely from PHB.St and PHB.TSP (weighing 0.25–0.35 g) were subjected to a test in which they were directly submerged in 100 mL of water for 5 days. Every 24 h, the material was removed from the container, lightly dried, and its weight measured. The test was performed in triplicate, and the swelling degree (SD%) was calculated using eq 1, where *W*_f_ is the swollen weight and *W*_i_ is the dry weight.^48^

1 $$\mathrm{SD(\%)}\,\;=\,\;\frac{W_{f}-W_{i}}{W_{i}}\times \mathrm{100}$$

## 3 Results and Discussion

### 3.1 Nutrient Release in Water

Figure 1 shows the release profiles of nutrients K and N (as K^+^ and NO_3_^–^) from both microspheres (recognized by the suffix “II”) and microcapsules (suffix “III”). The KNO_3_ salt used as a source of nutrients is highly soluble (with a solubility coefficient of ∼320 g/L at 20 °C), so any delay in its release is significant for fertilizer applications. In all microparticle formulations, the highest solubility coefficient of KNO_3_ (at 30 °C) was used, contributing to the phenomenon known as ″burst release″ (a rapid initial release within a specific time range). Despite this, the tablets released nutrients gradually overall. For K^+^ release, microparticles covered with the PHB.TPS matrix (Figure 1c) showed a faster burst release, whereas those with PHB.St (Figure 1a) delayed K^+^ release more effectively. The drying nozzle type also influenced the release profile, i.e., tablets containing microspheres (II) released K^+^ more slowly in the initial hours. As noted by França et al.,^40^ microparticles with PHB.St exhibited superior performance in NO_3_^–^ release (Figure 1b).

In contrast, PHB.TPS microparticles released 100% within 120 min (Figure 1d). During the initial hours, microspheres (II) exhibited a more sustained NO_3_^–^-release than microcapsules (III). The 3-fluid nozzle on Spray Dryers typically produces capsule structures, where nutrients or active ingredients are contained in the core, while a polymeric matrix forms the shell.^44^ However, the microcapsules (III) did not demonstrate greater efficiency than microspheres (II) in delaying the nutrient release as it was expected, excepted for the PHB.St/CNF^+^.KNO_3_III that prolonged NO_3_^–^ release the longest, behavior affected by incorporating positively charged CNF. Further mathematical modeling of the water release results helped to clarify the ion behavior within each matrix type.

### 3.2 Modeling

Tables 3 and 4 show the results of the software modeling evaluation for K^+^ and NO_3_^–^ release, respectively. For both nutrients, the Zero-order model demonstrated the lowest *r* ^2^ values across all the materials, indicating that factors beyond simple dissolution over time influenced the release mechanism. The First-order model showed an increase in *r* ^2^, suggesting that nutrient release was linked to incorporation into a porous matrix. This implies that the release rate was proportional to the amount of nutrients remaining in the matrix.

**Table 3:** Coefficient of Determination (*r*_adjusted_^2^) from Different Kinetics Models Fitted to Potassium Release from Composites Applied as EEFs

	release mechanism model for K^**+**^ release (*r*_adjusted_^2^)						
composites	Zero-order	First-order	Higuchi	Hixson-Crowell	Hopfenberg	Korsmeyer-Peppas	Peppas-Sahlin
PHB/St/CNF^+^.KNO_3_ II	0.6792	0.9651	0.9877	0.9264	0.9616	0.9909	0.9945
PHB/St/CNF^‑^.KNO_3_ II	0.6677	0.9442	0.9863	0.8922	0.9386	0.9882	0.9957
PHB/St/CNF^+^.KNO_3_ III	0.7666	0.9457	0.9663	0.9205	0.9406	0.9911	0.9962
PHB/St/CNF^‑^.KNO_3_ III	0.6886	0.9901	0.9791	0.9710	0.9891	0.9830	0.9963
PHB/TPS/CNF^+^.KNO_3_ II	0.7930	0.9972	0.9695	0.9910	0.9975	0.9862	0.9987
PHB/TPS/CNF^‑^.KNO_3_ II	0.6395	0.9848	0.9854	0.9571	0.9832	0.9856	0.9956
PHB/TPS/CNF^+^.KNO_3_ III	0.5472	0.9895	0.9856	0.9701	0.9884	0.9862	0.9994
PHB/TPS/CNF^‑^.KNO_3_ III	0.8870	0.9940	0.9816	0.9783	0.9930	0.9961	0.9994

**Table 4:** Coefficient of Determination (*r*_adjusted_^2^) from Different Kinetics Models Fitted to Nitrate Release from Composites Applied as EEFs

	release mechanism model for NO_3_^–^ release (*r*_adjusted_^2^)						
composites	Zero-order	First-order	Higuchi	Hixson-Crowell	Hopfenberg	Korsmeyer-Peppas	Peppas-Sahlin
PHB/St/CNF^+^.KNO_3_ II	0.7394	0.9886	0.9606	0.9627	0.9874	0.9724	0.9904
PHB/St/CNF^‑^.KNO_3_ II	0.7353	0.9892	0.9408	0.9689	0.9881	0.9519	0.9899
PHB/St/CNF^+^.KNO_3_ III	0.7997	0.9801	0.9330	0.9565	0.9781	0.9592	0.9814
PHB/St/CNF^‑^.KNO_3_ III	0.8228	0.9687	0.9256	0.9790	0.9873	0.9573	0.9731
PHB/TPS/CNF^+^.KNO_3_ II	0.7870	0.9820	0.9241	0.9671	0.9824	0.9473	0.9790
PHB/TPS/CNF^‑^.KNO_3_ II	0.7606	0.9893	0.9503	0.9672	0.9882	0.9650	0.9902
PHB/TPS/CNF^+^.KNO_3_ III	0.6413	0.9792	0.9375	0.9782	0.9860	0.9367	0.9898
PHB/TPS/CNF^‑^.KNO_3_ III	0.8152	0.9844	0.9168	0.9885	0.9889	0.9493	0.9773

Glycerol was used in TPS to enhance its thermoplastic properties by reducing the granular structure, resulting in a more homogeneous material. Although the starch was not fully plasticized, it was evident that the granule size dispersed within the PHB phase decreased, resulting in a more porous matrix (Figure 2a,b). On average, the *r* ^2^ values for the First-order model were higher in samples containing TPS, a trend more pronounced K^+^ release. SEM images supported this finding, illustrating nutrient dispersion into a more porous matrix (Figure 2e,i) and showing the matrix structure after nutrient release (Figure 2f,j). Formulations with CNF^–^ and microspheres (Figure 2c) exhibit greater homogeneity than those with microcapsules or TPS (Figure 2e–i), which appear more irregular and porous, with PHB.TPS (Figure 2f) matrices exhibiting greater postrelease deterioration than PHB.St. Similarly, the SEM images for CNF^+^ displayed comparable characteristics (Supporting data – Figure S1).

**Figure 2:**
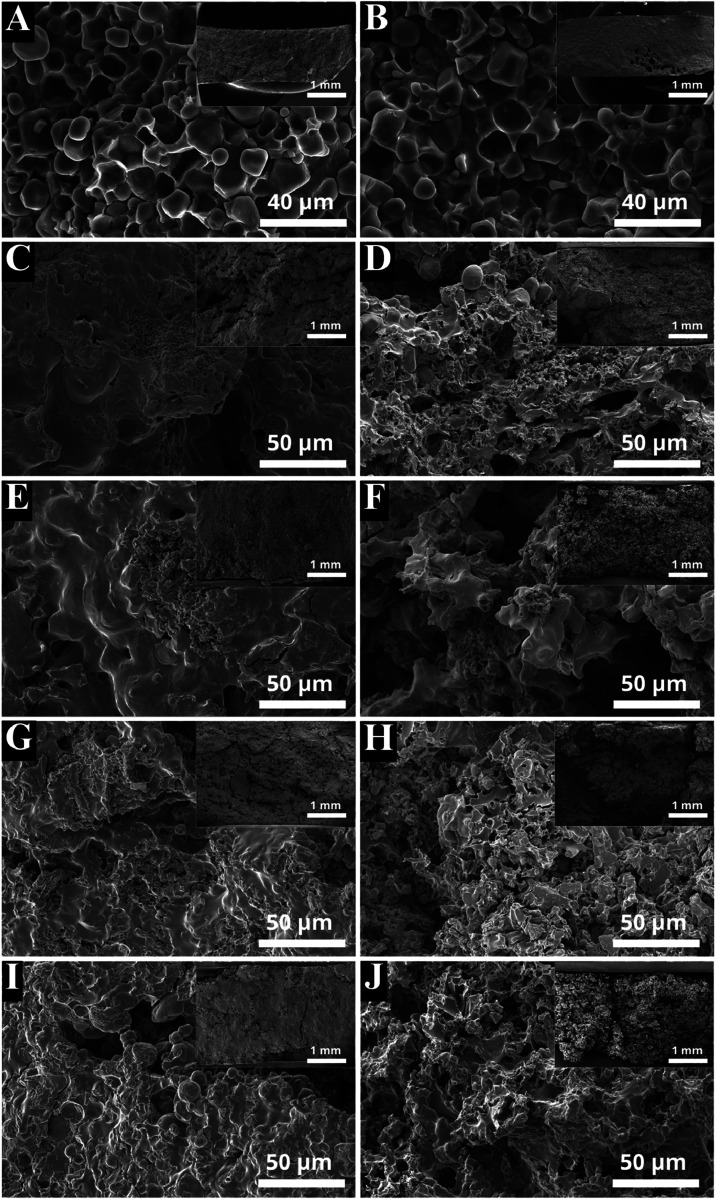
SEM image of the pure (a) PHB.St and (b) PHB.TPS and of the PHB/St/CNF^–^.KNO_3_ II, PHB/TPS/CNF^–^.KNO_3_ II, PHB/St/CNF^–^.KNO_3_ III and PHB/TPS/CNF^–^.KNO_3_ III composites (c, e, g, i) before and (d, f, h, j) after the nutrient release in water.

The Higuchi, Hixson-Crowell, and Hopfenberg models showed high *r* ^2^ for most composites (*r* ^2^ > 0.9000), suggesting that the release mechanism of dispersed particles within the matrix was primarily diffusion-driven. For the Higuchi model, *r* ^2^ values were higher for K^+^ release in all samples. Since this model assumes that the matrix dissolution or swelling does not contribute to the release mechanism, it can be inferred that the K^+^ diffusion is facilitated by its size and structure. The Hixson-Crowell model showed slightly higher *r* ^2^ values for NO_3_^–^ release and TPS materials. This model suggests that the release rate is governed more by matrix dissolution than by diffusion and is better suited for monodisperse materials and spheroidal particles. Therefore, the findings indicate that matrix dissolution significantly releases NO_3_^–^ and TPS-based materials, implying a more monodisperse system (Figure 2e,i, images with lower magnification).

The Hopfenberg model describes nutrient release as occurring primarily through surface erosion of the matrix (whether in a film, cylinder, or sphere) and also shows higher *r* ^2^ values for TPS-containing materials, particularly for NO_3_^–^ release. This is due to NO_3_^–^ an ion that encounters steric hindrance from its interactions with the matrix structure, explaining why models not solely reliant on matrix diffusion produce higher *r* ^2^ values. Additionally, as illustrated in Figure 2d–j, the surface changes in the matrix were observed after the water release, indicating surface erosion without complete dissolution.

The Korsmeyer-Peppas and Peppas-Sahlin empirical models provided the best fit for the experimental data, with the highest *r* ^2^ values (*r* ^2^ > 0.9800). Both models describe nutrient release from swelling and nonswelling polymer systems, with the Korsmeyer-Peppas model further indicating whether the release mechanism follows Fickian diffusion based on the release exponent (*n*). In terms of model adequacy, *r* ^2^ values were higher for K^+^ release, especially in tablets containing CNF^+^. As shown in Table 5, *n* values for all materials ranged from above 0.43 to below 0.89, suggesting anomalous release behavior in cylindrical samples.^37^ This behavior implies that diffusion and swelling influence the release mechanism, consistent with *r* ^2^ results observed for the other evaluated models. For NO_3_^–^ release, the n values were slightly higher, indicating a more significant deviation from Fickian diffusion. Additionally, for samples containing CNF^–^, the n values for K^+^ release were lower than NO_3_^–^ release, while the inverse was observed for materials with CNF^+^. This suggests that when ions are electrostatically bound to the matrix, diffusion has a more pronounced effect on the release mechanism than swelling. Although we have not conducted rheological characterizations regarding the viscoelasticity of the materials, the swelling test and the thermal characterization (DSC) help to understand that there is molecular mobility in the polymer chains used here as the matrix (Section 3.3).

**Table 5:** Release Exponent (*n*) from the Korsmeyer-Peppas Model for the Release Mechanism of the Composites Applied as EEFs

composites	Korsmeyer-Peppas model	
	potassium (K^+^) release	nitrate (NO_3_^–^) release
	N	*n*
PHB/TPS/CNF^‑^.KNO_3_ III	0.479	0.650
PHB/TPS/CNF^‑^.KNO_3_ II	0.525	0.596
PHB/St/CNF^‑^.KNO_3_ III	0.548	0.647
PHB/St/CNF^‑^.KNO_3_ II	0.535	0.587
PHB/St/CNF^+^.KNO_3_ II	0.540	0.581
PHB/St/CNF^+^.KNO_3_ III	0.603	0.630
PHB/TPS/CNF^+^.KNO_3_ II	0.600	0.626
PHB/TPS/CNF^+^.KNO_3_ III	0.620	0.544

Finally, the Peppas-Sahlin model was employed to elucidate the diffusional and relaxational mechanisms underlying the anomalous release of nutrients. This model accounts for the combined effects of Case II transport (polymer relaxation and swelling) and Fickian diffusion. Figure 3 illustrates the nutrient release curves of the PHB/TPS/CNF^+^.KNO_3_ III (a) and PHB/TPS/CNF^–^.KNO_3_ III (b) composites, demonstrating how closely they align with the predictions of the Peppas-Sahlin model. The release curves of the other composites exhibit a similar pattern, both for K^+^ and NO_3_^–^ release (Table S4 – Supporting Data).

**Figure 3:**
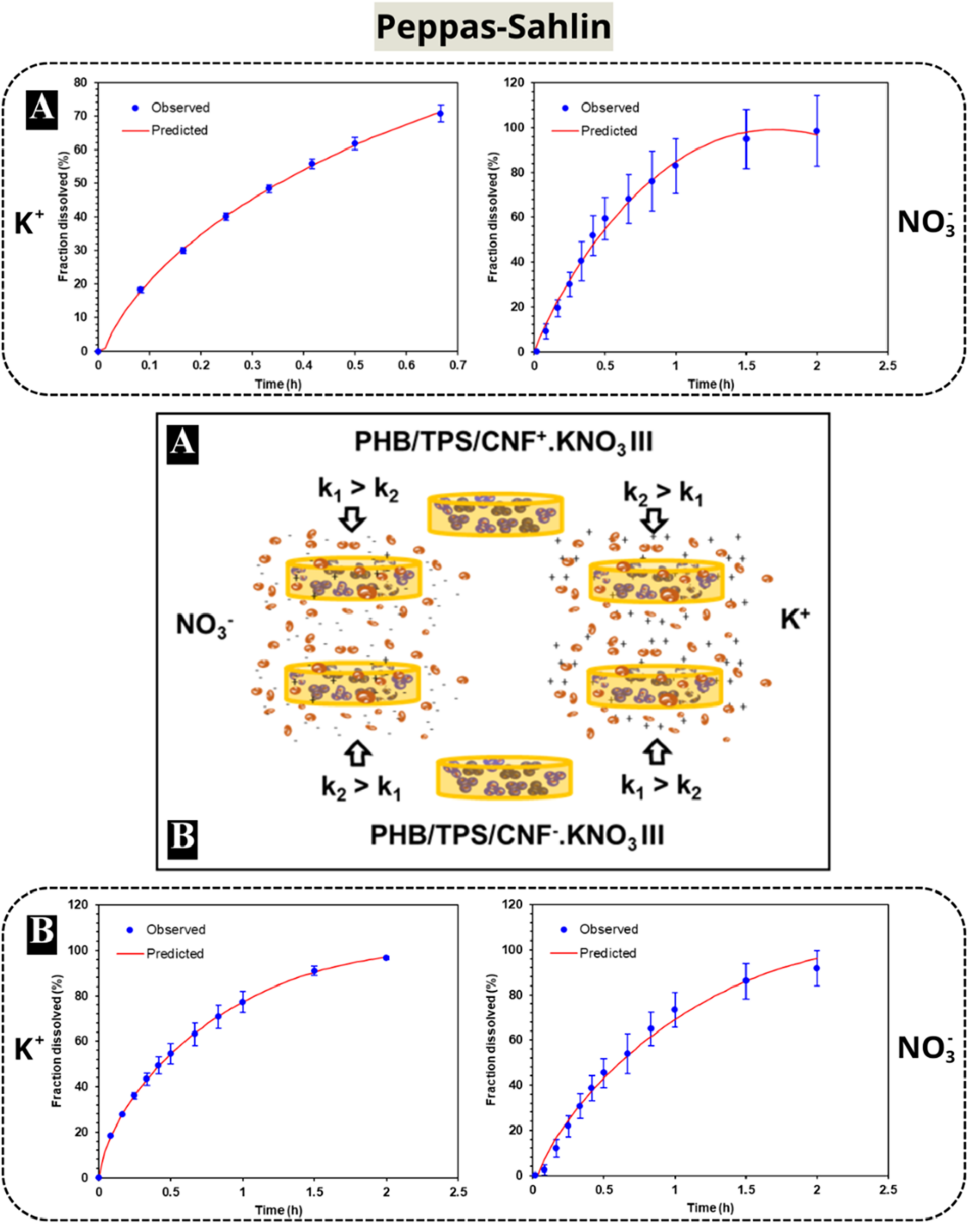
Release curves observed and predicted by the Peppas-Sahlin model for the PHB/TPS/CNF^+^.KNO_3_ III (a) and PHB/TPS/CNF^–^.KNO_3_ III (b) composites and scheme of the contribution of diffusional (*k*_1_) and relaxational (*k*_2_) constants on nutrient release correlated to the functionalized-CNF.

Table 6 shows the *k*_1_ values representing diffusional contribution and the *k*_2_ indicating the polymeric chain relaxation contribution.^39^ When *k*_2_ yielded negative results, the diffusion contribution (*k*_1_) predominated during the release process. Analyzing all composites containing functionalized CNF, we compared the polymer chain diffusion and relaxation constants derived from the Peppas-Sahlin model. The data revealed that the contributions of these constants varied depending on the nutrient type (K^+^ or NO_3_^–^) and the functional group on the cellulosic surface (CNF^–^ or CNF^+^). For instance, PHB.TPS/CNF^–^.KNO_3_ III, which contained negatively charged CNF (CNF^–^), exhibited a high diffusion constant (*k*_1_ = 104.487) and a low relaxation constant (*k*_2_ = −27.268) during the release of the positively charged nutrient K^+^. Conversely, when PHB.TPS/CNF^–^.KNO_3_ III released NO_3_^–^, the diffusion constant was lower (*k*_1_ = −16.639), and the relaxation constant was higher (*k*_2_ = 85.689), corroborating the results obtained from the Korsmeyer-Peppas model. This trend was observed for all microcapsules and microspheres, although the difference between the *k*_1_ and *k*_2_ constants was smaller for the microspheres (Table 6).

**Table 6:** Parameters from the Peppas-Sahlin Model for the Release Mechanism of the Composites Applied as EEFs[Table-fn t6fn1]

composites	Peppas-Sahlin model					
	potassium (K^+^) release			nitrate (NO_3_^–^) release		
	*k*_1_	*k*_2_	*r* ^2^	*k*_1_	*k*_2_	*r* ^2^
PHB/TPS/CNF^‑^.KNO_3_ III	104.487	–27.268	0.9994	–16.639	85.689	0.9773
PHB/TPS/CNF^‑^.KNO_3_ II	91.224	–21.433	0.9956	32.1	31.122	0.9902
PHB/St/CNF^‑^.KNO_3_ III	92.123	–22.464	0.9963	–81.315	152.781	0.9731
PHB/St/CNF^‑^.KNO_3_ II	68.67	–14.995	0.9957	94.797	–26.969	0.9899
PHB/St/CNF^+^.KNO_3_ II	73.696	–13.402	0.9945	42.707	19.502	0.9904
PHB/St/CNF^+^.KNO_3_ III	40.645	13.65	0.9962	32.74	23.26	0.9814
PHB/TPS/CNF^+^.KNO_3_ II	15.933	52.362	0.9987	27.194	36.491	0.979
PHB/TPS/CNF^+^.KNO_3_ III	3.709	80.79	0.9994	122.625	–37.905	0.9898

1 *k*_1_ – diffusional constant, *k*_2_ – polymeric chain relaxation constant, *r* ^2^ – coefficient of determination.

These findings suggest that the release of the positively charged nutrient K^+^ relies more heavily on the diffusion constant (*k*_1_) to exit matrices with negative charges (CNF^–^), while the polymeric chain relaxation (*k*_2_) is more influential for release from matrices with positive charges (CNF^+^). The opposite was also observed; the release of negatively charged nutrient NO_3_^–^ depended more on the diffusion constant (*k*_1_) to release from positive matrices (CNF^+^) and on the relaxational constant (*k*_2_) for release from negative matrices (CNF^–^), Figure 3. It is noteworthy that the AIC values are consistent with the *r* ^2^ values, being mostly lower values for the Korsmeyer-Peppas and Peppas-Sahlin models (Tables S1, S2 – Supporting data).

### 3.3 Swelling Properties of Matrices

Figure 4 presents the mass and swelling degree (SD) data of the tablets composed of PHB.St and PHB.TPS matrices over the 5-day swelling test period. From these results, it was observed that the matrix containing starch (PHB.St) exhibited a higher swelling degree (13.93% after 5 days) compared to the matrix containing thermoplastic starch (PHB.TPS), which reached 8.75% in the same period. Notably, after just 24 h of immersion (Day 1) – a time point at which the majority of K^+^ and NO_3_^–^ ions were already released – an SD of 4.69% was recorded for the PHB.St matrix and 7.57% for the PHB.TPS matrix. As previously shown in the SEM images (Figure 2), the PHB.TPS matrix is slightly more porous due to the reduced size of the starch granules, which facilitates greater water permeation during the initial hours of the test. Although the overall SD values for both matrices remain low, the data provide further evidence that ion release occurs not only via diffusion but also through matrix relaxation. Moreover, the negative glass transition temperature (*T*_g_) values were observed for the PHB.St and PHB.TPS matrices indicate that, under normal environmental conditions, the polymer exists in a more flexible and soft state (Table S3 – Supporting data).

**Figure 4:**
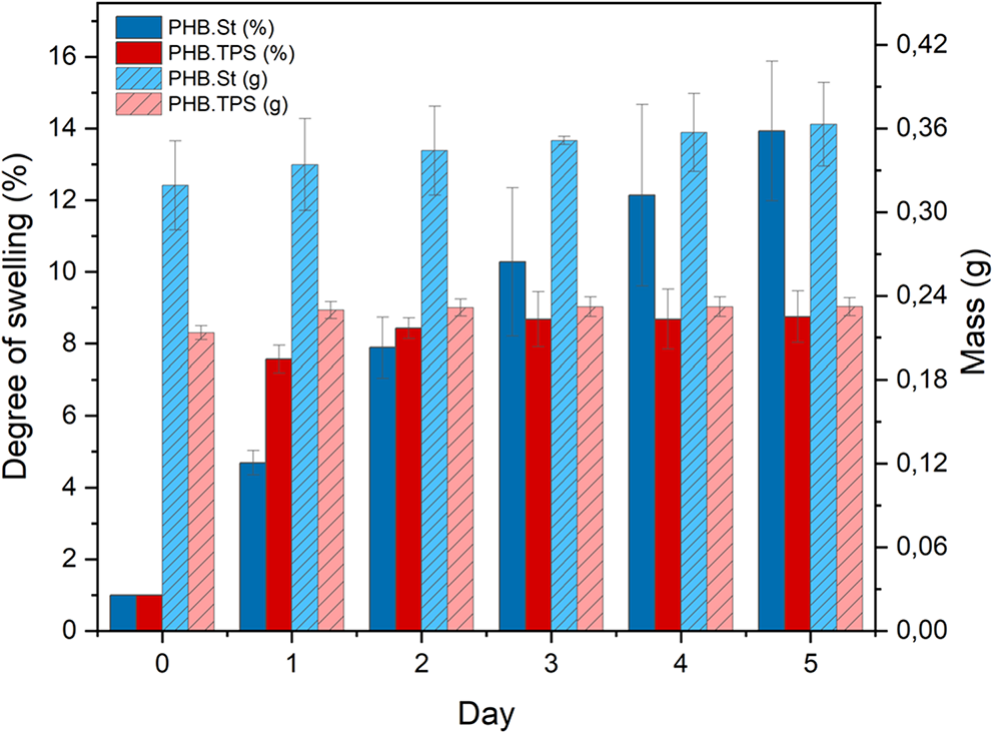
Mass (g) and swelling degree (SD%) of PHB.St and PHB.TPS tablets in water at 25 °C.

Swelling tests were not conducted on the composite tablets containing CNF and KNO_3_-based fillers, as the mass loss associated with the release behavior influenced the results. Analyses of matrices with pure KNO_3_ (data not shown) revealed that the simultaneous swelling and rapid release of KNO_3_ resulted in final mass values lower than the initial ones.

## 4 Conclusion

This study provides valuable mechanistic insights under controlled laboratory conditions and expands upon previous research on soil nutrient release and biodegradability.^40^ EEFs based on modified CNF, PHB, and St (or TPS) exhibited distinct K^+^ and NO_3_^–^ release profiles, influenced by CNF surface charge, polymer matrix composition, and microparticle morphology (spheres vs capsules). The Higuchi, Hixson-Crowell, and Hopfenberg models show good agreement with experimental data, highlighting the roles of diffusion, dissolution, and matrix erosion. However, the semiempirical Korsmeyer-Peppas and Peppas-Sahlin models yielded the best fits, indicating anomalous transport mechanisms driven by a combination of diffusion and polymer chain relaxation. Release behavior depended on CNF functionalization. In CNF^–^ systems, K^+^ release was driven by diffusion, while NO_3_^–^ was controlled by polymer relaxation. In contrast, CNF^+^ systems showed the opposite trend, with NO_3_^–^ release dominated by diffusion and K^+^ release influenced by relaxation mechanisms. Differences in microparticle morphology were also observed. Microcapsules exhibited faster nutrient release, while microspheres, with simpler structures, promoted more gradual release, particularly for K^+^. These findings demonstrate that tailoring CNF charge and matrix composition enables modulation of nutrient release rates. This strategy offers a promising route to improve nutrient use efficiency in agriculture and supports the development of biodegradable, high-performance EEFs.

## Supplementary Material



## Notes

Additional SEM images of CNF^+^ based composites, Akaike Information Criterion (AIC), thermal information about the matrices, release curves observed and predicted by the Korsmeyer-Peppas and Peppas-Sahlin models for all the formulations, and ANOVA for the burst release of the nutrients and at the end point (PDF)
