# Supplementary material for: The Role of Cellulose Charge and Matrix Composition on KNO3‑Nutrient Release Kinetics and Mechanisms
Source: ACS Omega. 2026 Feb 19;11(8):13084–93. doi: 10.1021/acsomega.5c06715 (PMC12961548; doi:10.1021/acsomega.5c06715)
Supplement: Supplementary file 1 [file ao5c06715_si_001.pdf]

The role of cellulose charge and matrix composition on KNO_3-nutrient release kinetics and mechanisms

Débora França[a], Sahmira Bianchi[a,b], Roselena Faez[a,b]*

[a] Laboratory of Polymeric Materials and Biosorbents, Federal University of São Carlos, UFSCar, Rod. Anhanguera, km 174, Araras, SP, 13600970, Brazil

[b] Graduate Program in Materials Science and Engineering, University of São Paulo, USP- FZEA, Pirassununga, SP, 13635900, Brazil

*Prof. Dr. Roselena Faez - faez@ufscar.br
Lab-MPB - Laboratório de Materiais Poliméricos e Biossorventes
Departamento de Ciências da Natureza, Matemática e Educação
Universidade Federal de São Carlos
Rod. Anhanguera, km 174 - SP-330, P.O. BOX 153
13600-970 Araras - São Paulo – Brasil
Phone: +55 (19) 3543-2946
*Sahmira Bianchi - sahmirabianchi@usp.com
*Débora França - deborafransa@gmail.com

SEM images of CNF^+ based composites

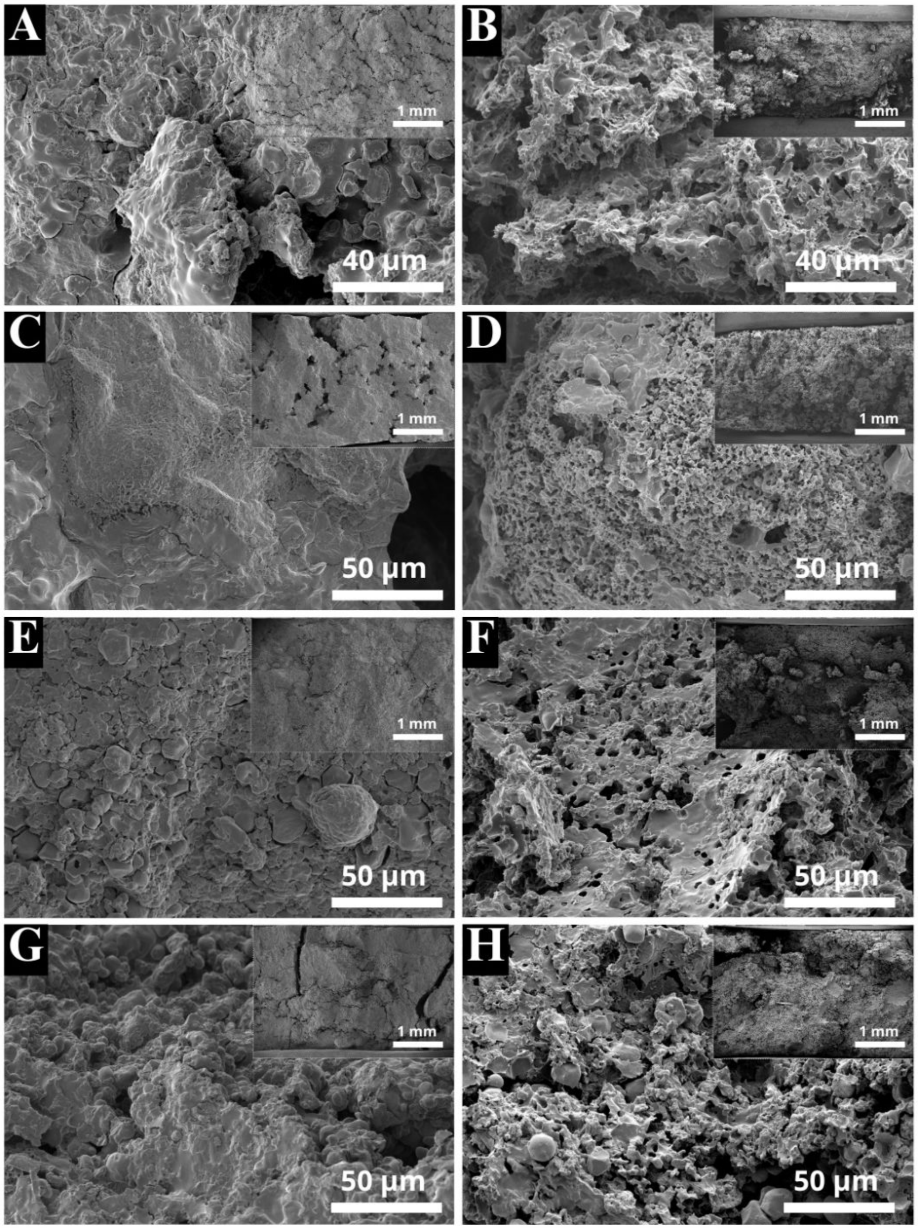

Fig S1. SEM image of the pure the PHB/St/CNF^+.KNO_3 II, PHB/TPS/CNF^+.KNO_3 II, PHB/St/CNF^+.KNO_3 III and PHB/TPS/CNF^+.KNO_3 III composites (a,c,e,g) before and (b,d,f,h) after the nutrient release in-water.

As observed in the composites with CNF^-, the formulations based on microspheres (Fig. S1 a,c) exhibit greater homogeneity compared to those formed with microcapsules (Fig. S1 e,g),

which may have contributed to a slower nutrient release. Similarly, consistent with the characteristics of the composites with CNF^-, the formulations containing TPS appear more irregular and/or porous, as evident in the lower magnification images (Fig. S1 c,e). Additionally, the images obtained after the release test reveal that the PHB.TPS-based matrix experienced more significant deterioration compared to the PHB.St-based matrix, another factor influencing the nutrient release rate.

Akaike Information Criterion (AIC)

Table S1. AIC from different kinetics models fitted to K^+ release from composites applied as EEFs

Composites	Release Mecanism model for K^+ release (AIC)						
	**Zero-order**	**First-order**	**Higuchi**	**Hixson-Crowell**	**Hopfenberg**	**Korsmeyer-Peppas**	**Peppas-Sahlin**
**PHB/St/CNF⁺.KNO₃ II**	93.4 ± 1.3	66.2 ± 3.7	54.2 ± 3.2	75.3 ± 3.2	68.2 ± 3.8	51.4 ± 1.9	46.2 ± 0.3
**PHB/St/CNF⁻.KNO₃ II**	90.9 ± 0.5	69.4 ± 1.4	52.6 ± 1.4	77.3 ± 1.0	71.5 ± 1.4	51.7 ± 0.4	40.3 ± 1.6
**PHB/St/CNF⁺.KNO₃ III**	86.1 ± 6.1	59.0 ± 14.2	63.1 ± 14.2	64.6 ± 13.3	60.6 ± 14.5	45.8 ± 12.1	36.6 ± 10.0
**PHB/St/CNF⁻.KNO₃ III**	96.3 ± 3.6	53.1 ± 6.4	63.9 ± 3.9	66.8 ± 4.3	55.1 ± 6.4	61.5 ± 7.2	44.7 ± 0.8
**PHB/TPS/CNF⁺.KNO₃ II**	92.6 ± 2.6	40.7 ± 4.9	69.1 ± 5.0	54.5 ± 5.1	40.4 ± 4.0	61.0 ± 2.6	32.9 ± 4.2
**PHB/TPS/CNF⁻.KNO₃ II**	97.9 ± 0.3	59.7 ± 2.4	59.4 ± 0.9	72.3 ± 1.6	61.8 ± 2.4	60.0 ± 0.3	46.5 ± 1.2
**PHB/TPS/CNF⁺.KNO₃ III**	50.7 ± 0.5	26.8 ± 2.9	36.2 ± 0.7	37.6 ± 1.5	28.8 ± 2.8	23.7 ± 12.3	7.4 ± 9.9
**PHB/TPS/CNF⁻.KNO₃ III**	102.2 ± 2.7	54.7 ± 9.3	57.5 ± 12.3	69.2 ± 3.6	56.8 ± 9.2	58.3 ± 11.3	23.4 ± 6.6

Table S2. AIC from different kinetics models fitted to NO_3^- release from composites applied as EEFs

Composites	Release Mechanism model for NO_3^- release (AIC)						
	**Zero-order**	**First-order**	**Higuchi**	**Hixson-Crowell**	**Hopfenberg**	**Korsmeyer-Peppas**	**Peppas-Sahlin**
**PHB/St/CNF⁺.KNO₃ II**	92.3 ± 5.0	52.6 ± 8.0	69.7 ± 3.1	71.5 ± 4.3	64.6 ± 8.0	66.5 ± 3.2	53.4 ± 5.3
**PHB/St/CNF⁻.KNO₃ II**	94.5 ± 3.1	54.6 ± 7.2	76.4 ± 2.3	68.6 ± 2.7	56.6 ± 7.2	74.8 ± 2.4	56.9 ± 1.7

**PHB/St/CNF⁺.KNO₃ III**	88.0 ± 3.6	59.3 ± 4.1	74.8 ± 5.5	68.9 ± 4.6	61.4 ± 4.1	69.9 ± 4.9	60.2 ± 6.8
**PHB/St/CNF⁻.KNO₃ III**	93.8 ± 5.1	67.2 ± 18.7	83.3 ± 6.2	62.2 ± 19.2	58.4 ± 16.5	77.0 ± 8.0	71.8 ± 9.6
**PHB/TPS/CNF⁺.KNO₃ II**	92.0 ± 1.4	61.8 ± 7.7	79.3 ± 6.8	69.2 ± 1.6	62.8 ± 6.0	75.9 ± 6.2	61.0 ± 16.2
**PHB/TPS/CNF⁻.KNO₃ II**	92.4 ± 0.8	50.7 ± 10.0	73.5 ± 4.2	66.3 ± 6.5	52.7 ± 10.0	70.2 ± 2.6	53.7 ± 9.7
**PHB/TPS/CNF⁺.KNO₃ III**	102.3 ± 4.6	65.4 ± 12.2	81.3 ± 3.4	65.6 ± 10.0	62.1 ± 10.0	82.3 ± 3.5	61.1 ± 4.2
**PHB/TPS/CNF⁻.KNO₃ III**	92.9 ± 5.0	63.5 ± 2.8	83.5 ± 1.2	57.8 ± 8.2	58.4 ± 7.8	78.4 ± 8.3	66.5 ± 10.5

The AIC values obtained for the evaluated models, both for K^+ (Table S1) and NO_3^- (Table S2) release, further support our previous discussion based on the adjusted Coefficient of Determination (r^2). For most of the analyzed samples, the Peppas-Sahlin model consistently demonstrated the lowest AIC values, reaffirming its superior fit in describing the release mechanism of the EEFs samples. This alignment between AIC and r^2 strengthens the robustness of our findings, indicating that the Peppas-Sahlin model remains the most appropriate choice for characterizing the nutrient release behavior in this study.

Thermal Analysis

Table S3. Thermal events observed by DSC for the matrices (PHB.St and PHB.TPS)

		Endothermic events (°C)	
Matrix	**Tg (°C)**	**1st endothermic event**	**2nd endothermic event**
**PHB.St**	-12.6	177.4	302.6
**PHB.TPS**	-6.5	171.5	305.6

When the glass transition temperature (Tg) of a polymeric material is below 0 °C, it indicates that under normal environmental conditions, the polymer will exist in a more flexible and soft state, rather than being hard and brittle. This supports our findings, which suggest that polymer chain relaxation facilitates nutrient release.

Observed vs. predicted release curves from semi-empirical models

Table S4. Release curves observed and predicted by the Korsmeyer-Peppas and Peppas-Sahlin model for the PHB/TPS/CNF^{+}.KNO_3 III and PHB/TPS/CNF^{-}.KNO_3 III composites

Samples	Korsmeyer-Peppas<br>$F = k_{KP}\ t^{n}$		Peppas-Sahlin<br>$F = k_1\ t^{m} + k_2\ t^{2m}$	
	K^+	NO_3^-	K^+	NO_3^-
PHB.St/CNF$^+$.KNO_3 II	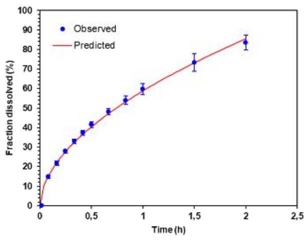	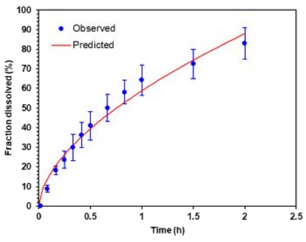	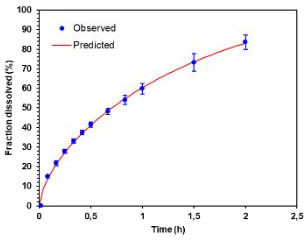	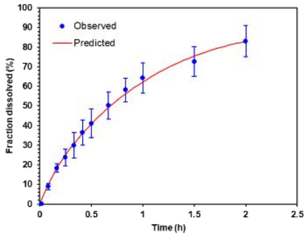
PHB.St/CNF$^-$.KNO_3 II	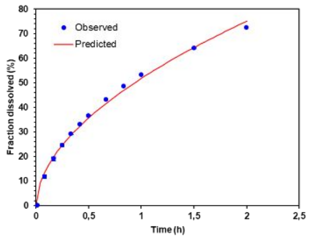	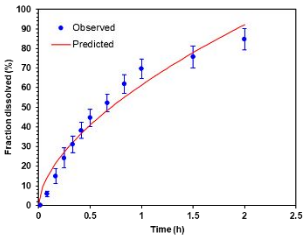	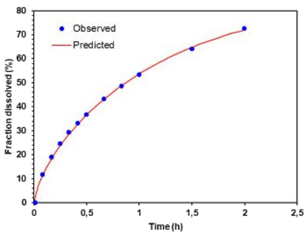	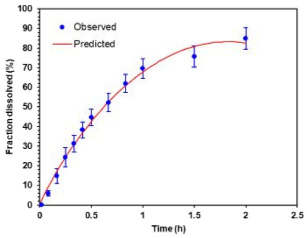
PHB.St/CNF$^+$.KNO_3	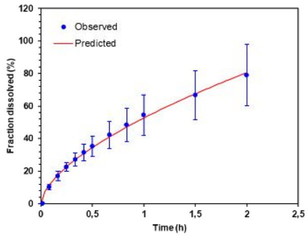	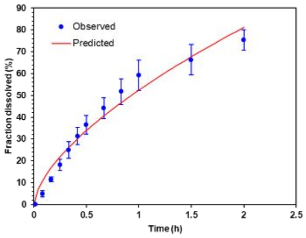	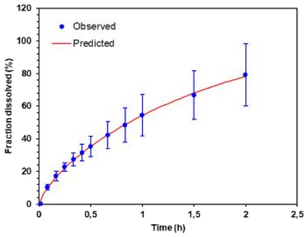	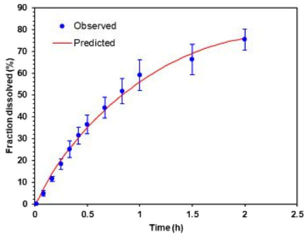
PHB.St/CNF$^-$.KNO_3 III	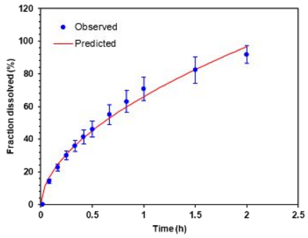	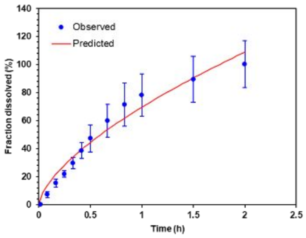	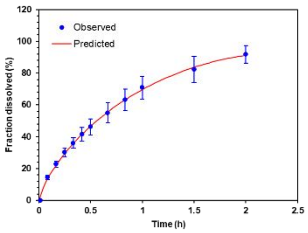	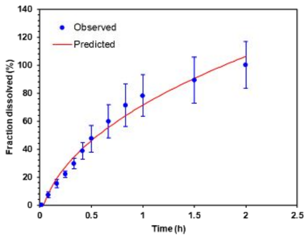

PHB.TPS/CNF^{+}.KNO_3 II

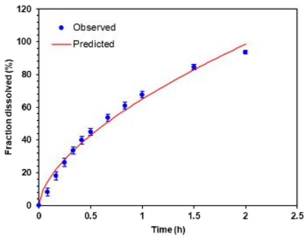

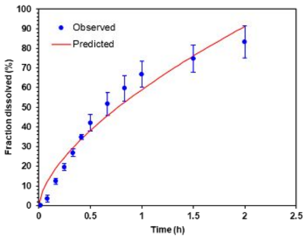

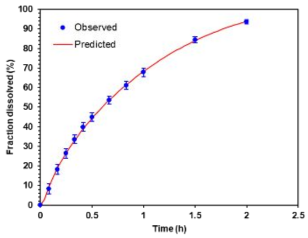

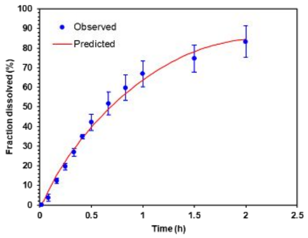

PHB.TPS/CNF^{-}.KNO_3 II

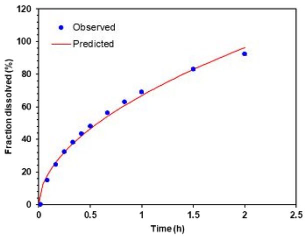

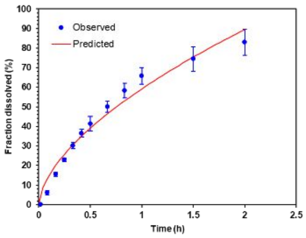

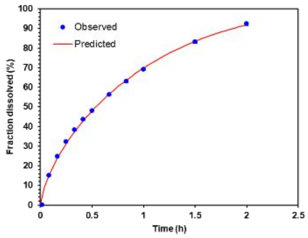

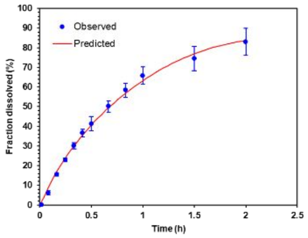

PHB.TPS/CNF^{+}.KNO_3 III

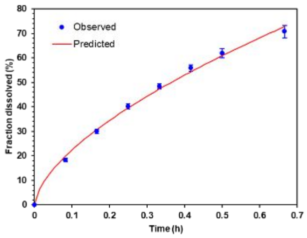

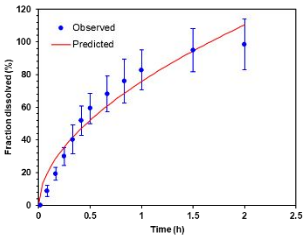

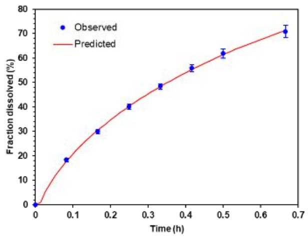

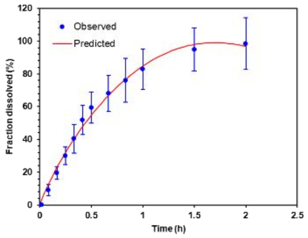

PHB.TPS/CNF^{-}.KNO_3 III

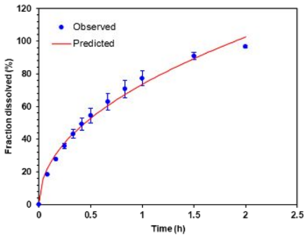

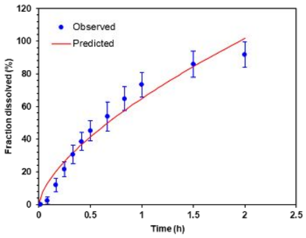

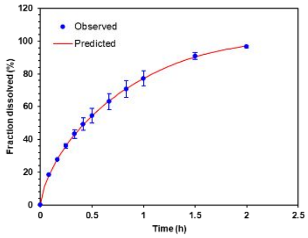

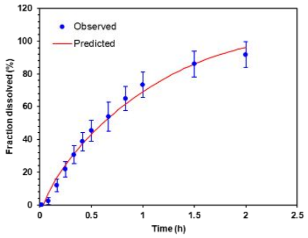

ANOVA

By comparing the burst release with the release at the end-point, we can confirm a significant difference in the initial nutrient release behavior between the matrices, as indicated by the high F-statistic value and the very low p-value (muche smaller then 0.05). At the end-point, this difference is less pronounced than during the burst release, as evidenced by the respective F-statistic and p-value.

Table S5. ANOVA for the burst release of the nutrients and at the end point.

SUMMARY - Burst release

Groups	*Count*	*Sum*	*Average*	*Variance*
K^+ -PHB/St/CNF^+.KNO_3 II	3	44.84237	14.94746	0.740416
NO_3^- -PHB/St/CNF^+.KNO_3 II	3	26.76959	8.923198	2.849064
K^+ -PHB/St/CNF^-.KNO_3 II	3	35.08929	11.69643	0.418336
NO_3^- -PHB/St/CNF^-.KNO_3 II	3	17.59893	5.86631	2.079164
K^+ -PHB/St/CNF^+.KNO_3 III	3	30.94979	10.3166	2.883034
NO_3^- -PHB/St/CNF^+.KNO_3 III	3	14.66904	4.88968	1.772797
K^+ -PHB/St/CNF^-.KNO_3 III	3	43.33872	14.44624	1.667075
NO_3^- -PHB/St/CNF^-.KNO_3 III	3	21.46793	7.155976	4.116213
K^+ -PHB/TPS/CNF^+.KNO_3 II	3	24.95073	8.316909	6.660068
NO_3^- -PHB/TPS/CNF^+.KNO_3 II	3	10.7015	3.567166	3.191455
K^+ -PHB/TPS/CNF^-.KNO_3 II	3	45.32113	15.10704	0.233851
NO_3^- -PHB/TPS/CNF^-.KNO_3 II	3	18.18729	6.06243	1.198715
K^+ -PHB/TPS/CNF^+.KNO_3 III	3	54.7232	18.24107	0.582994
NO_3^- -PHB/TPS/CNF^+.KNO_3 III	3	26.85736	8.952453	11.17325
K^+ -PHB/TPS/CNF^-.KNO_3 III	3	55.26079	18.42026	0.016871
NO_3^- -PHB/TPS/CNF^-.KNO_3 III	3	7.280106	2.426702	5.00413

ANOVA

Source of Variation	*SS*	*df*	*MS*	*F*	*P-value*	*F crit*
Between Groups	1148.149	15	76.54327	27.4672	4.6E-14	1.99199
Within Groups	89.17488	32	2.786715			
Total	1237.324	47				

SUMMARY - End Point

Groups	*Count*	*Sum*	*Average*	*Variance*
K^+ -PHB/St/CNF^+.KNO_3 II	3	250.5904	83.53014	13.82322

NO_3^- -PHB/St/CNF$^+$.KNO_3 II	3	248.925	82.97501	64.2934
K^+ -PHB/St/CNF$^-$.KNO_3 II	3	217.5163	72.50543	0.036373
NO_3^- -PHB/St/CNF$^-$.KNO_3 II	3	254.3683	84.78944	29.94469
K^+ -PHB/St/CNF$^+$.KNO_3 III	3	237.3292	79.10973	359.8921
NO_3^- -PHB/St/CNF$^+$.KNO_3 III	3	226.18	75.39332	22.45453
K^+ -PHB/St/CNF$^-$.KNO_3 III	3	275.4852	91.82839	29.30552
NO_3^- -PHB/St/CNF$^-$.KNO_3 III	3	300.5729	100.191	282.1384
K^+ -PHB/TPS/CNF$^+$.KNO_3 II	3	280.646	93.54868	1.299567
NO_3^- -PHB/TPS/CNF$^+$.KNO_3 II	3	249.8005	83.26683	65.63603
K^+ -PHB/TPS/CNF$^-$.KNO_3 II	3	276.9016	92.30053	0.634768
NO_3^- -PHB/TPS/CNF$^-$.KNO_3 II	3	249.1077	83.03592	45.67521
K^+ -PHB/TPS/CNF$^+$.KNO_3 III	3	298.3563	99.4521	1.386459
NO_3^- -PHB/TPS/CNF$^+$.KNO_3 III	3	295.3127	98.43756	245.5992
K^+ -PHB/TPS/CNF$^-$.KNO_3 III	3	290.0294	96.67647	0.438259
NO_3^- -PHB/TPS/CNF$^-$.KNO_3 III	3	275.2489	91.74963	61.1121

ANOVA

Source of Variation	*SS*	*df*	*MS*	*F*	*P-value*	*F crit*
Between Groups	3367.749	15	224.5166	2.93565	0.005148	1.99199
Within Groups	2447.34	32	76.47936			
Total	5815.089	47				
